# A Mechanism of Synaptic Clock Underlying Subjective Time Perception

**DOI:** 10.3389/fnins.2019.00716

**Published:** 2019-07-09

**Authors:** Bartosz Jura

**Affiliations:** Nalecz Institute of Biocybernetics and Biomedical Engineering, Polish Academy of Sciences, Warsaw, Poland

**Keywords:** dopamine, ecology, flicker fusion effect, reward-based learning, synaptic tagging and capture, time perception

## Abstract

Temporal resolution of visual information processing is thought to be an important factor in predator-prey interactions, shaped in the course of evolution by animals’ particular ecology. Here I show that light can be considered to have a dual role of a source of information, which guides motor actions, and an environmental feedback for those actions. I consequently show how temporal perception might depend on feedback-based behavioral adaptations realized in the nervous system through activity-dependent synaptic plasticity. I propose an underlying mechanism of synaptic clock, with every synapse having its characteristic time unit, determined by the persistence of memory traces of synaptic inputs, which is used by the synapse to tell time, and postulate the existence of a specific brain-wide distribution of synaptic clocks with different time units. The present theory offers a simple, testable link between the fields of neurobiology of memory, time perception and ecology, which may account for numerous experimental findings, including the interspecies variation in the temporal resolution and the properties of subjective time perception in humans, specifically the variable speed of perceived time passage, depending on emotional or attentional states or tasks performed.

## Introduction

From everyday life we know that subjective perception of time is not constant and time might appear to flow slower or faster, depending, for example, on the emotional or attentional state or task being performed (Dirnberger et al., 2012; Hagura et al., 2012; Lake et al., 2016). However, the exact physiological mechanisms that may underlie this phenomenon remain elusive.

It is generally recognized that time perception is not a sensory modality on its own, but a temporal dimension is inherent to perception of sensory stimuli, execution of motor tasks as well as all aspects of cognitive processing. Accordingly, the recent view is that there is no single, centralized clock in the brain, dedicated to performing any operations related to timing, regardless of the nature of a task. Instead, imaging data show that distributed brain areas are involved in time-keeping, which suggests that specialized areas have their own built-in mechanisms, that may vary among systems (Buhusi and Meck, 2005; Díaz-Mataix et al., 2014; Muller and Nobre, 2014; Gouvêa et al., 2015). Consequently, timing has been proposed to be a direct result of the dynamical nature of neural activity and plasticity of the nervous system leading to the evolution of neural activity over time. Time could thus be encoded in the progression of neural activity patterns through a state space (Paton and Buonomano, 2018). Importantly, timing of intervals in the sub-second range is thought to involve different brain regions than timing of longer ones (Lewis and Miall, 2003).

The studies of perception of temporal intervals during exposure to stimuli coming from a single sensory modality, that is, for example, during observation of a dynamically changing visual stimulus, suggest that estimates of duration depend on the number of changes (i.e., bits of information) which are subjectively perceived in a unit time, rather than reflecting an objective duration of the interval (Kanai et al., 2006). Namely, when a higher number of distinct visual events is detected over a temporal window of a given length, the perception of this interval is more fine-grained and it seems to last longer. Consequently, the corresponding time flow seems to slow down, giving an impression of time dilation. Conversely, a lower number of events detected results in a more course-grained perception of the interval, which therefore seems to last shorter and the time flow appears to speed up. Accordingly, it has been proposed that estimates of duration correspond to the expenditure of neural energy used to process a stimulus (Eagleman and Pariyadath, 2009). Therefore, it appears that, in simple tasks limited to a single sensory modality, provided that duration of the interval which is to be timed is not compared to any reference duration values, such of stimuli coming from a different sensory modality or such stored in memory, the number of changes detected can be studied as an equivalent of subjective time perception.

Within the visual modality, a phenomenon which allows to directly measure the number of changes detected in a unit time and, moreover, compare it in different animal species, is related to a property of the visual system termed Critical Frequency of Flicker Fusion (CFF). CFF determines the highest frequency of a flickering light stimulus, or the corresponding minimum inter-event interval, which allows to distinguish subsequent light impulses as separate events. Frequencies of flicker above that value cause the impulses to be fused and perceived as a continuous stimulus. The exact value of fusion frequency depends on multiple factors, e.g., light wavelength or intensity or background lighting conditions, with the CFF usually specifying the highest threshold value found in any condition, representative for a particular species (Umeton et al., 2017). It is assessed using electroretinography (Frank, 2000) or, in animals, in behavioral paradigms in which animals are rewarded for responding to a stimulus the flicker of which they can perceive (Lisney et al., 2011), and, in humans, using psychophysical tests in which subjects report the critical frequency (Hardy, 1920). The phenomenon of CFF has been attributed to a temporal integration of signal in the retina and downstream pathways of information processing (Nardella et al., 2014). It is used as a measure of temporal resolution of visual information processing, representing a maximum number of bits of information that can be detected in a unit time. Higher CFF values correspond to higher resolutions, as more information is absorbed over a temporal window of the same length and more rapid changes in a stimulus can be detected, as opposed to lower CFF values, when information is integrated over longer time windows. It follows that the value of CFF might be considered a measure of subjective time perception as resulting from the processing of visual input.

**FIGURE 1:**
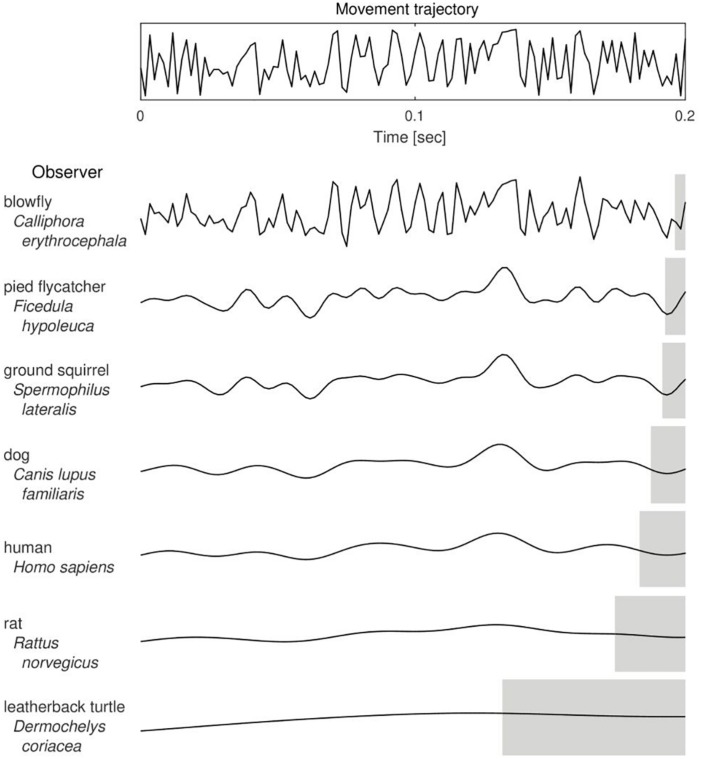
The putative effect of Critical Frequency of Flicker Fusion value on the trajectory of a moving object as perceived by different animal species. With a decrease in the number of bits of visual information detected in a unit time (from the top to the bottom of the table), a perceived trajectory of the actual movement (upper panel) is more course-grained and hence the estimated position of the observed object is less accurate. Gray shaded areas indicate lengths of the corresponding temporal windows of information integration, that is, intervals of duration equal to 1/CFF. Curves were drawn based on CFF data taken from and referenced by Healy et al. (2013), except for the blowfly (Autrum, 1949) and the pied flycatcher (Boström et al., 2016).

The value of CFF was shown by Healy et al. (2013) to be linked with body size and mass-specific metabolic rate of animals. As body size affects the inertia and thus maneuverability of animals, it was therefore proposed that CFF and, consequently, temporal perception, is an important factor in predator-prey interactions. Namely, in a model situation, given a sufficient level of their maneuverability and speed of movements, predators with higher CFF are able to precisely detect changes in the movement trajectory of a fast-moving prey and follow its steps and, conversely, prey with higher CFF might avoid being caught and escape predation by being able to see predator’s movements in a “slow-motion” and react earlier (Figure 1). Therefore, the level of maneuverability determines whether it is worth to invest in a high-resolution visual system and hence it might have been a significant evolutionary constraint upon shaping the CFF in different species. What follows is that the CFF itself can be studied as a functional trait which influences predator-prey interactions and which is shaped in an adaptive evolutionary-ecological game (Schmitz, 2017).

Since perception of a passage of time is tightly related to the detection of change, and change can be detected only due to memory, it suggests the underlying neurophysiological mechanisms of time perception to be in some way related to those of memory. However, so far, it has remained unclear what type of memory or which neural underpinnings are critically involved. Classic models referring to a notion of working memory, sought primarily in network dynamics, are not doing well in explaining more recent, subtle empirical data (Muller and Nobre, 2014). It is thought, however, that memory is stored primarily due to changes in synaptic connections, with the Long-Term Potentiation (LTP) being a central model for learning and memory (Bliss and Lømo, 1973). Here I use this very notion and show that a simple theoretical mechanism of temporal perception can be derived directly from the mechanisms of synaptic plasticity, particularly the phenomenon of synaptic tagging (Frey and Morris, 1997). I show that the interspecies variability of CFF, when considered from the ecological perspective, can be related to the processes of activity-dependent synaptic plasticity seen as feedback-based behavioral adaptations performed by the nervous system. Consequently, I propose a mechanism of synaptic clock, with every synapse having its characteristic time unit, determined by the persistence of transient memory traces of synaptic inputs, which constitutes a form of temporal filter and is used by the synapse to tell time. CFF can be then shown to be a special case of the general rule, which imposes limitations on the temporal resolution of information processing. I postulate the existence of a brain-wide distribution of synaptic clocks with different time units which depend on, and increase with, the level of abstraction of information processed by a given synapse, or the distance of a synapse from the sensory and motor regions. The variable speed of perceived time passage can be then explained as a result of functioning of different synaptic clocks which measure time in different units. The present study thus offers a testable theory which may account for numerous experimental findings.

## A Dual Role of Light

### CFF in Predator-Prey Interactions and a Dual Role of Light

In a situation of predator-prey encounter an impulse of light can be considered (1) a source of information which guides animals’ motor actions, and (2) an environmental feedback for those actions.

**FIGURE 2:**
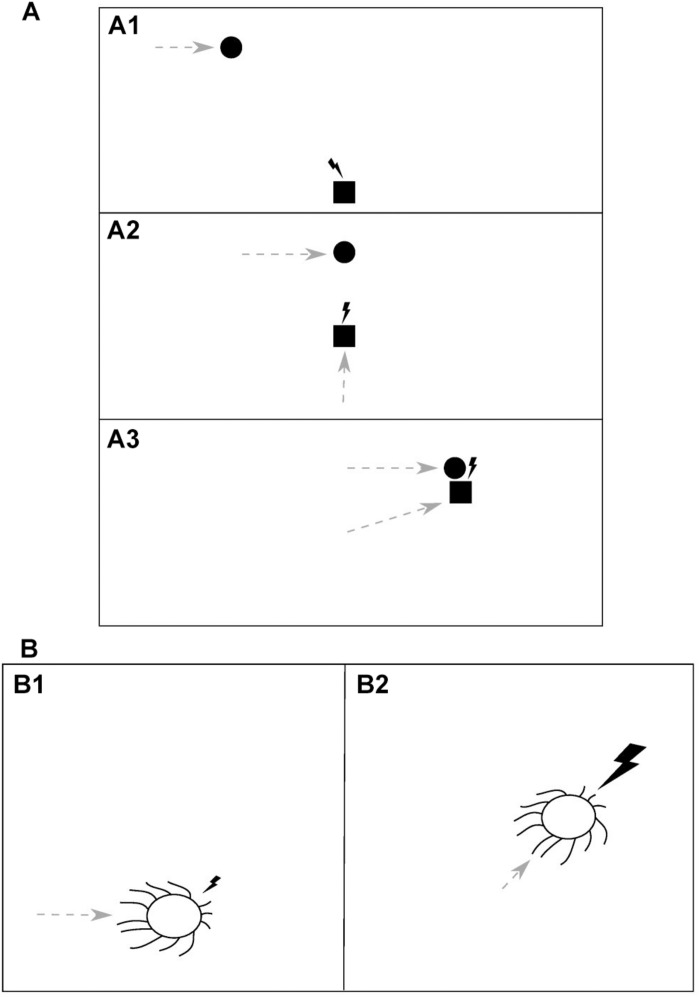
The dual role of light as a source of information, which guides motor actions, and an environmental feedback for those actions. **(A)** In a predator-prey interaction (i.e., prey pursuit) an impulse of light (lightning bolt) serves as **(A1)** a sensory cue which indicates the position and movement of the prey (circle) to the predator (square) and guides its subsequent motor action, **(A2)** a feedback for the predator’s action (dashed gray arrow) and another cue, allowing the predator to update its information about the prey, and **(A3)** a feedback for the predator’s action, delivered along with (or being a substitute of) the prey itself. The animals’ temporal resolution of visual information processing, in order to be advantageous, should be proportional to time intervals between information-guided motor actions and the corresponding feedbacks delivery. Thus, it should be adequate to the speed with which the relative spatial position of the animals changes, that is, the relative speed of movement of the predator and the prey. **(B)** A direct manifestation of light being an environmental feedback for motor actions is found in the phototaxis, where it is utilized, for example by cyanobacteria, in photosynthesis. An impulse of light, falling from a certain direction, is a cue which guides a motor action of the organism **(B1)**. The subsequent light impulse of higher intensity is a reward for that action, delivered after the organism makes a move toward the light **(B2)**. This example appears to constitute an upper limit for the temporal resolution of information processing as it should be proportional to the speed of light and hence will be determined only by the rate of an underlying molecular processing.

Consider an idealized situation of a predator chasing its prey, as depicted in Figure 2A. When the predator (square) initially detects the moving prey (circle) due to the visual information received with the first impulse of light (lightning bolt) (Figure 2A1), it starts to move itself (gray dashed arrows) and after every step it receives an update on the prey’s position, in the form of another impulse of light, and so it can update its predictions and adjust its previous movement trajectory (Figure 2A2). Eventually, when the predator makes its final move and catches the prey, it gets the prey itself as a feedback for its motor action along with the last impulse of light (Figure 2A3). Thus, an analogy can be drawn here between the light and the prey, both serving as environmental feedback used to evaluate the predator’s actions.

Therefore, the rate of processing of visual information (i.e., the CFF) should be adequate for the relative speed of movement of the predator and the prey, in order to allow the predator to catch its target (or, the other way round, the prey to escape predation). Specifically, the time interval between two impulses of light that can be distinguished as two separate sensory stimuli should be proportional to the interval between a visual information-guided action and the subsequent feedback for that action (in a given step of the “simulation,” e.g., the one in Figure 2A2). If this interval is too short and the predator detects another sensory stimulus before its spatial position relative to that of the prey changes, then such information is redundant and energy used to process it will be wasted. Conversely, if the interval is too long and the predator fails to detect another sensory stimulus after the relative position changes, it will not know what was the outcome of its action. It follows that the averaged inter-event interval of CFF should be fine-tuned in order to give organisms a survival advantage. Now, we will consider the constraints that limit the value of that interval.

The shortest possible interval between a motor action and a feedback is determined by the highest possible speed of predator-prey interaction and information transfer, which is the speed of light. Therefore, the highest possible value of CFF, giving an organism a virtually perfect (i.e., infinitely high) temporal resolution of information processing, should be developed when light itself is a “prey” and feedback for motor actions, for example when it is utilized by an organism to produce nutrients in photosynthesis, which is actually the case in the phenomenon of phototaxis.

### A Direct Manifestation of the Dual Role of Light

Phototaxis is a phenomenon found both in prokaryotes and eukaryotes, in which organisms can move along a light gradient. It has been found, among others, in photosynthesizing organisms (for a review, see Jékely, 2009). It was demonstrated by Schuergers et al. (2016) that one of such organisms (unicellular cyanobacterium *Synechocystis* sp. PCC 6803) acts as a microlens and can precisely sense the direction of light and move directly toward its higher intensity, in order to obtain, as an effect, more light energy for the photosynthesis. Thus, in this organism light serves both as a sensory cue which guides its motor actions and a “prey,” being an environmental feedback for those actions (Figure 2B). As soon as the organism makes a move toward the light (Figure 2B1), a brighter ray of light can reach its thylakoid photoreceptors and nutrients be produced in photosynthesis (Figure 2B2). Therefore, in order to allow the organism to react immediately to any changes in the lighting direction, the temporal resolution of sensory information processing should be proportional to the speed of light in the medium. However, since molecular signals of the cascade triggered by a sensory input and leading to the execution of a motor action cannot decay faster than with the speed of light, the actual resolution will be lower and limited by the decay rate of that molecular trace left by the input.

In this case of phototaxis, the goal of organism’s actions is itself used as a sensory cue which guides the actions. Therefore, the relation between the sensory information and the goal is constant and hence the information-guided actions do not need to be evaluated and adjusted according to their outcomes. It follows that the molecular signal might decay spontaneously, immediately after it triggers a motor action and there is no need to sustain it until a feedback is delivered. This appears to hold also in other cases where movements are simple reflexes and where the relation between sensory information and goals does not change, also when the relative speed of movement of an organism and its goals is lower than in the case of phototaxis. For example in chemotactic organisms, which can detect and move along the concentration gradient of various chemical substances (for a review, see Wadhams and Armitage, 2004). There, similarly, a target substance is usually used as an action-guiding sensory cue and the relation between them is constant (or dependent only on a temporary internal state of an organism, e.g., its current metabolic demands). Therefore, input signals also do not need to be sustained until a feedback is delivered (or except only to detect instantaneous changes in the concentration gradient). It follows that there are no constraints on the minimal time of persistence of input signals and therefore the value of temporal resolution does not have a strong upper bound. However, the situation is different in organisms which can use neutral information with changing relation to goals to guide their motor actions, which is the case in organisms with a nervous system.

## Activity-Dependent Synaptic Plasticity as Feedback-Based Behavioral Adaptations

Evolution of the nervous system has separated the channels of motor output from the channels of sensory input with additional, intermediate stages of information processing. It enabled organisms to use various types of information with complex relation to goals to guide their motor actions. Importantly, they can use neutral information, and the relation of information to goals can change. The learning of abstract relations between neutral information and goals, based on the outcomes of actions triggered by such information, is performed through the activity-dependent plasticity of the nervous system, which leads to behavioral adaptations. The most well-studied aspect of such plasticity is the activity-dependent synaptic plasticity (Sweatt, 2016). The view of behavioral adaptation as the primary function of synaptic plasticity is supported by the features of nervous system. Namely, any act of synaptic transmission eventually affects the execution of motor actions, due to the polarity of synaptic connections and directionality of transmission of effective signals in the nervous system. Ultimately, sensory inputs are received through some dedicated channels, and motor outputs are generated through others. The existence of recurrent connections does not contradict this directionality, as any circuit of recurrently connected cells must eventually receive an extrinsic input and project an output. Any activity-dependent change in synaptic weights should therefore be beneficial and not harmful for the organism as a whole, that is, it should result in actions the performance of which is adjusted to given situations.

This utilitarian nature of the activity-dependent synaptic plasticity is reflected in the three-factor learning rule, which suggests that not only Hebbian co-activation of pre- and post-synaptic cells is required for an enduring synaptic modification to occur, but also the influence of neuromodulation, with dopamine (DA) playing a key role in this respect in the mammalian brain (Lisman et al., 2001; Reynolds and Wickens, 2002). DA has been shown to be required for the protein synthesis-dependent late phase of LTP (L-LTP) in distributed brain areas (Otani et al., 2015; Broussard et al., 2016). DA might exert its permissive effect on plasticity even when it is delivered with a delay (Lisman and Grace, 2005) and, moreover, it can bidirectionally affect the synaptic change [i.e., allowing for L-LTP or late-phase Long-Term Depression (L-LTD; Huang et al., 2004), or, depending on the concentration, transforming a potential LTP into LTD and *vice versa* (Thivierge et al., 2007)], and thus it can be viewed as an internal substitute of environmental feedbacks for synaptic activity.

Importantly, since organisms live in spatially extended environments, environmental feedbacks are never delivered immediately after a synaptic activity. Sometimes, a relevant feedback might be very distant in time from an information-guided action. As a result, organisms need to adjust their behavior based on delayed feedbacks (so-called “distal reward” problem), and different types of information-guided actions tend to have different temporal distance to the action-evaluating goals.

## Derivation of a Mechanism of Synaptic Clock From a Notion of Synaptic Tagging

### The Persistence of Memory Traces of Synaptic Inputs

**FIGURE 3:**
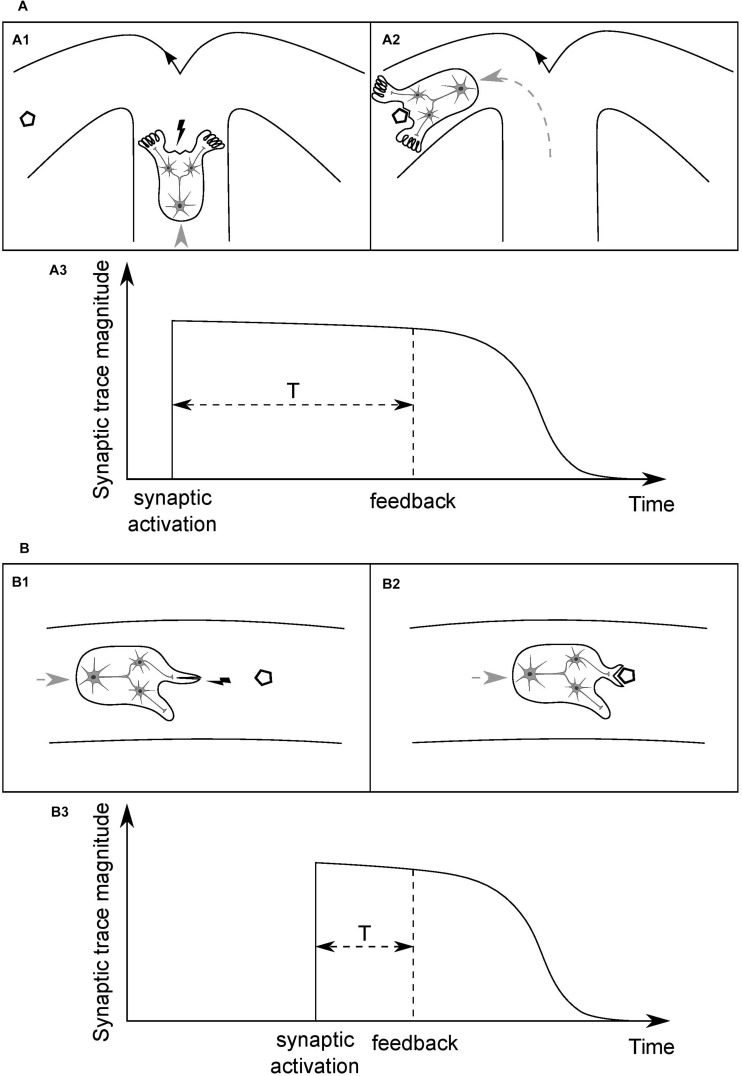
Time course of the persistence of memory traces of synaptic inputs, and its dependence on the type of information processed by a given synapse. **(A) (A1)** Cartoon depicts an idealized organism with a simple neural circuit, moving at a constant speed in a medium and having limited energy resources, the input neuron of which detects specific combinations of visual cues in the environment (crossroad) and activates one of the postsynaptic output neurons, which change direction of the organism’s movement. In order to be modified appropriately, based on the outcome of its activity, an activated synapse has to stay tagged until position of the food (polygon), or end of the empty arm, is reached **(A2)**. **(A3)** A time course of the magnitude of a memory trace of synaptic input, serving to tag the synapse. The required duration of persistence of the synaptic trace will determine a characteristic time unit for this type of synapse. **(B) (B1)** An organism analogous to that in **(A)** but the input neuron of which detects food portions being in close proximity and activates one of the output neurons, which leads either to opening of the mouth or execution of some other, inadequate motor action. Here, the feedback for a motor action is delivered within a shorter time window than in **(A)** and thus synapses have to stay tagged for a shorter period of time **(B2)**. **(B3)** This synaptic trace should decay faster than that in **(A)**, and thus it will determine a shorter time unit for this type of synapse. Different types of synapse will have different, characteristic time units, shaped as the averaged temporal distance of a particular type of information-guided actions to goals. Actual values of the time units will be shaped by organisms’ speed of movements and, more generally, rate of interactions with their surroundings. The synapse depicted in **(A)** might be an analogy to a synapse of a hippocampal pyramidal neuron and the one in **(B)** to a striatal medium spiny neuron’s.

Consider an idealized organism (O1) with a simple neural circuit, as depicted in Figure 3A1, and the synaptic connections between a presynaptic input neuron and two postsynaptic output neurons. Assume that the input neuron is a higher-level unit which detects a specific type of places in the environment, based on a combination of visual cues (crossroad), and the output units innervate limbs which can change direction of the organism’s movement, turning the body either left or right. Assume further that the organism is moving forward at a constant speed in a medium and has limited energy resources, sufficient only to reach the nearest energy supply (polygon). When the organism arrives at the crossroad then, in order to survive, it needs to enter the arm with the food and to do so it has to perform a motor turn in the appropriate direction. Initially, weights of the synaptic connections are random. Then, if the sensory unit does not activate the correct motor unit and the organism enters the empty arm, it will not reach the food and die. If the organism does perform a correct motor turn and receives an environmental feedback for that action in the form of the food reward (Figure 3A2), and it leads to the strengthening of the synapse the activity of which has led to this beneficial outcome, the survival chances of the organism will increase. However, if the organism performs the correct action but the activated synapse is not strengthened after receiving the feedback, the chances of performing a correct action when being next time in similar situation, and thus of survival, will stay on the same level as with a random set of synaptic weights.

Since the feedback delivery is not immediate and, on the other hand, synaptic activity can be transient and short-lasting, the synaptic input needs to leave a tag on the synapse so that it can be reinforced when a relevant feedback is delivered. From now on, we will refer to this type of synapse-specific tag, left on a synapse by a synaptic input, as the synaptic *trace*.

### Duration of the Synaptic Trace

If such a trace does not persist for long enough and decays before the organism can reach the food reward, then, even after performing a correct action and receiving the feedback, the synapse will not be modified and the final result will be the same as there was no synaptic trace at all. Conversely, if duration of the trace is too long, the tagged synapse can be altered due to further environmental feedbacks, unrelated to the action triggered by a particular input stimulus and activity of the synapse. Eventually, the modification of the synaptic weight can get reversed and, as a net effect, activity of the synapse in the given situation will leave no mark in the network and, yet again, the result will be analogous to the lack of plasticity. However, when duration of the trace is properly adjusted and it persists just until the environmental feedback for the given synaptic activity is delivered, the connection can be reinforced effectively. Subsequently, when the organism finds itself again in a similar situation, it can make a right decision, based on its previous experience, on how it behaved and what outcome that action had brought.

Eventually, the only property that distinguishes an organism that will survive from other individuals is the duration of the synaptic trace being fine-tuned for the type of information processed in the given synapse, which allows for an effective act of synaptic plasticity to occur (Figure 3A3).

### Different Types of Synapse

Consider now another organism (O2), as depicted in Figure 3B1, analogous to the O1, and the synaptic connections between an input and two output neurons, the functions of which, however, differ from those of the O1. Here, the sensory unit detects food portions that the organism is approaching, and short-lasting activation of one of the motor units leads to opening of the mouth and thereby enables the organism to eat the food when it reaches its position (Figure 3B2). On the other hand, activation of the other motor unit results in execution of another, task-irrelevant action, e.g., moving a limb, and consequently, when the organism reaches the position of the food, the food will get bounced off its body and, as a result, it will die from a lack of energy. Therefore, for the type of synapse in this organism, the time interval between synaptic activation and delivery of relevant environmental feedback is shorter than in the case of the O1. It follows that the synaptic trace, in order to be advantageous, should also have shorter duration (Figure 3B3).

### Synapse Type-Specific Trace Duration

We argue that every synapse, belonging to a class of synapses processing a given type of information, develops a mechanism of synaptic trace of a specific duration, proportional to the expected value of time interval which separates synaptic activations from the delivery of relevant feedbacks.

### Synaptic CFF

The synaptic trace can be viewed as a short-lasting memory of the synaptic event that led to its formation and, consequently, the rate of trace decay can be viewed as a rate of forgetting about that event. Similarly, the fusion of sequential visual stimuli can be viewed as a result of short-lasting memory of single stimuli [also known as visual persistence (Hardy, 1920)] and the CFF can be viewed as a rate of forgetting about those stimuli. Therefore, by way of analogy, a synapse can be considered as a “sensory” unit, which receives external signals of a certain type and sustains memory of the input for a characteristic period of time, determining its temporal resolution of information processing. Within this time interval, the incoming inputs will be fused, and the same feedbacks will be applied to them, and thus the interval will constitute a synaptic “CFF.”

### Synaptic Clock

Since a default duration of such a synaptic trace is constant, it constitutes a time unit which can be used by the synapse to tell time. Hence, synapses might work as clocks and, since trace duration is synapse-specific, every synaptic clock will be telling time in its own unit.

### Synaptic Clocks and Anticipation

The formation of a synaptic trace, by an event itself neutral, can be viewed as an act of prediction that a feedback for that synaptic action will be delivered in the future, within a time interval specified by the trace duration. Thus, the functioning of synaptic clock, namely the decay of synaptic trace, can be viewed as a passive process of goal anticipation.

When an organism comprises multiple synapses, each of them processing a different type of information (e.g., a composition of the O1 and the O2), then every synapse will have a trace mechanism with a different individual time unit. However, goals pursued by an organism, having a significance for its survival, affect the organism as a whole and thus are common for all of its components. None of the organism’s synapses “knows” what goal is being pursued at the moment, and the only thing any of the tagged synapses “knows” is how far the goal is, or how fast it is being approached, as indicated by its individual time unit. Therefore, regardless of what the current goal is, the functioning of every synaptic clock indicates its own, expected time remaining to the goal. Consequently, the time interval-sensing function performed by synaptic clocks acting as temporal filters, as derived from our simple model, can be seen as implicit, and not relying on intentional, cognitively driven referring to the contents of memory and measuring a time interval that has passed since some event in the past. It stems rather from the continuous process of goal anticipation and thus is related to an event expected to occur in the future.

### Effect of Attention

Attention can be defined to be manifested through an increased activity rate of some neural population. Thus, attention leads to an elevated rate of stimulation of postsynaptic cells and, eventually, formation of an increased number of synaptic traces with high, initial magnitudes. As a result, a short-lasting memory of inputs processed by the stimulated synapses is stronger, relatively to that in other populations, and so a temporal perception will be more dependent on the pool of synapses in such a highly active region. Ultimately, the overall temporal perception of an organism might be reduced to the dynamics of synaptic clocks with different time units.

## A Global Distribution of Synaptic Clocks

### Synaptic Trace as a Dynamical Process

The mechanism of synaptic tag, as demonstrated originally within the framework of synaptic tagging and capture, shows that a weak synaptic stimulation can leave a protein synthesis-independent tag on the synapse, so that plasticity-related proteins (PRPs) synthesized non-locally in the cell body can find the synapses that were activated recently and secure their long-lasting modification (L-LTP; Frey and Morris, 1997).

Here, by synaptic “trace” we define more generally a transient (i.e., decaying), protein synthesis-independent state of a synapse, triggered by a synaptic input, in which it remains susceptible to the action of factors required for an enduring synaptic modification (as substantiated by L-LTP). That is, in other words, a transient “memory trace” of synaptic inputs [which might include a state of certain “malleability” of the synapse, due to a dismantling and reorganization of cytoskeleton elements within dendritic spines (Okamoto et al., 2009)]. We posit that an evolutionary pressure, as derived above, acts on the times of persistence of such a state of synapses, consequently adjusting them to the intervals between specific types of behavior and relevant feedbacks. This state might be not homogenous in terms of its specific dynamics and molecular nature across different synapses or brain regions, and thus be determined primarily by its function.

There is evidence that indeed the molecular nature of a tag might depend on the synapse (as it can depend on the process involved (i.e., LTP or LTD) or localization within a dendritic tree (i.e., basal or apical dendritic compartments in hippocampal CA1 neurons); Martin, 2002; Sajikumar et al., 2007; Okamoto et al., 2009). Consequently, the central mechanism of a synaptic clock may be implemented in different synapses by different molecular underpinnings. Moreover, different synaptic clocks within a cell will be presumably interrelated, due to intracellular signaling and diffusion of tagging molecules, especially within synaptic compartments (Sajikumar et al., 2007).

Synaptic tag is identified as an early phase of LTP (E-LTP), that is, an activity-dependent synaptic change which does not require protein synthesis, and which can be transformed into the late phase through an action of captured PRPs (Frey and Morris, 1997; Wang et al., 2010). It has been proposed to underlie a short-lasting, initial stage of long-term memory of everyday behavioral events (Ballarini et al., 2009; Wang et al., 2010). The same mechanisms and molecules have been implicated both in the synaptic tagging and LTP, notably, among others, the Ca^2+^/calmodulin-dependent protein kinase II (CaMKII) (Moncada et al., 2011; Lisman et al., 2012). In hippocampal neurons, synaptic tag has been shown to persist for about 0.5 h, as PRPs synthesized, or DA delivered, this long after a given synaptic event can effectively reinforce change of the recently activated synapses (Lisman et al., 2001; Ballarini et al., 2009). However, a molecular cascade triggered by a synaptic stimulation is shown to consist of multiple stages, all of which are needed for an LTP to occur and which, importantly, have different durations. For example, CaMKII autonomous activity [which is limited to single dendritic spines (Lee et al., 2009)] lasts only for about 1 min, whereas the state of its autophosphorylation persists for longer time periods (Lengyel et al., 2004). Therefore, based on observations like these, a synaptic trace, as we define it, can be reasonably seen as a dynamical process, starting with a synaptic input and comprising a sequence of molecular processing stages with varied time constants. Thus, the time unit of a synaptic clock should be proportional to a total duration of the corresponding molecular cascade but it might as well consist of multiple stages and be determined primarily by the effect this cascade has on the cell and circuit activity and, consequently, behavior.

### The Distribution and Action of Synaptic Clocks

An analogy for such sequential processing of information can be found in the visual system, where the short-lasting visual persistence of stimuli, being an early phase of information processing, is quickly transformed into a medium-term memory, which helps constructing an entire visual scene from a sequence of visual elements, viewed one by one (Melcher, 2001). Therefore, the other way round, the persistence of visual stimuli, determining the CFF, could be considered as being due to an initial stage of synaptic traces with very short time units.

**FIGURE 4:**
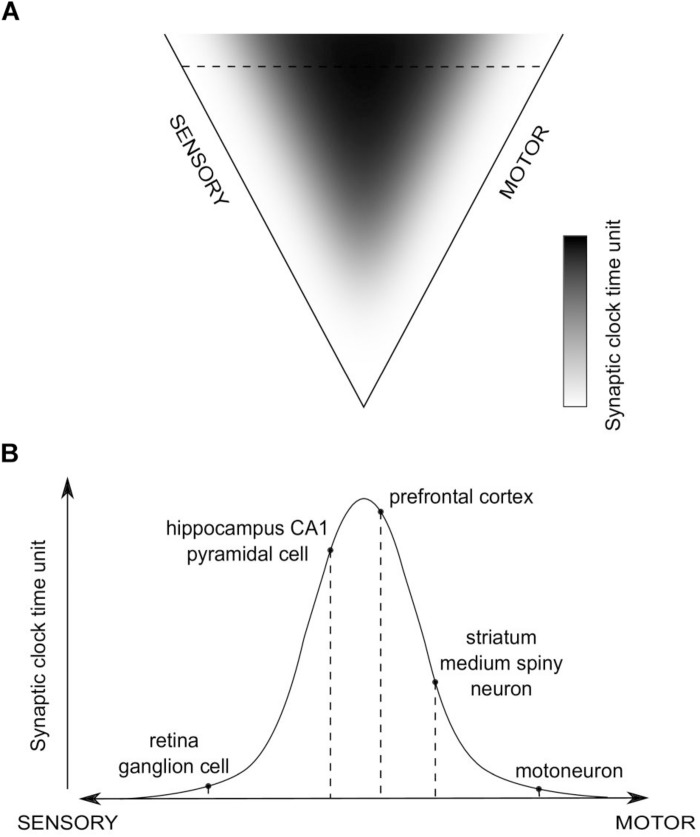
The hypothesized global distribution of synaptic clocks and their action affecting a perception of time passage. **(A)** The distribution of time units of different synaptic clocks, depending on their position on a sensory-motor continuum. Color-coded is a relative value of synaptic clock time unit, corresponding to the level of abstraction of information processed in a given synapse (black - large; white - small). **(B)** An example set of activated synapses with different time units of synaptic clocks, indicated in **(A)** as the horizontal dashed line. The pathway is going through the hippocampus, prefrontal cortex (PFC) and striatum. An overall temporal perception, resulting from the action of the corresponding set of synaptic clocks, might refer to a situation in which animal is performing a motor turn into a certain direction (action-selection - striatum; motor action - motoneuron) on a crossroad while navigating (visual processing - retina; spatial navigation - hippocampus) to a target location, as stored in the long-term reference memory (PFC).

Consequently, a global distribution of time units of synaptic clocks in the brain could be proposed, in relation to sensory and motor extrema (Figure 4). We hypothesize that values of the time units will positively correlate with the level of abstraction of information processed in a given synapse, which, in turn, is related to an average distance of the synapse from the sensory and motor regions. Then, when a purely sensory information is processed, or an automatic, reflex motor action is executed, without involvement of higher-order, associative brain areas, synaptic clocks with shorter time units will be triggered, which should result in more information processed in a unit time and the impression of a more fine-grained experience, as if the time flow was slowing down. On the other hand, when more abstract, for example spatial, information is processed, brain areas containing synaptic clocks with longer time units will be active, e.g., the hippocampus, which should result in fewer bits of information being processed in a unit time and the impression of time flow speeding up.

This distribution allows to make predictions, first, about the direction and magnitude of time perception distortions depending on the task performed and attention directed to different aspects of the task (e.g., sensory modalities involved), and, second, about interspecies differences in temporal perception, which should result from nervous systems having distributions of synaptic clocks with different values of the time units.

### The Issue of Timescale

The definition of synaptic trace that we employ here encompasses timescales traditionally associated with the persistence of synaptic tags, that is such ranging from minutes to hours, as demonstrated by Frey and Morris (1997) for hippocampal neurons, but it accounts also for shorter timescales, including the sub-second range specific for the visual CFF phenomenon (Healy et al., 2013). Such a presumed, very short-lasting synaptic traces could be seen as “degenerated” traces, which decay shortly after they are formed, and which can mediate actions of only those L-LTP-related factors that are already there at the synapse at the moment of trace formation [that is, for instance, of neuromodulators remaining in the extracellular space within synaptic junction, and/or intracellular PRPs produced due to some preceding, stronger stimulation through a converging pathway (Frey and Morris, 1997)].

It should be also stressed that a synaptic trace, as it is postulated here, although mediates actions of L-LTP-related factors, should be, at least in part, independent of those factors, namely, such a state of a synapse may persist for its characteristic period of time even after some PRPs have been already captured, or transition to a late phase of LTP has been already made.

### Synaptic Clocks and Allocation of Temporal Perception

Due to a threshold nature of synaptic tagging, one should expect that not all synaptic inputs will manage to leave a synaptic trace or affect the functioning of a synaptic clock. Consequently, perhaps only a subset of synapses within a given circuit will be affecting an ongoing temporal perception at a given moment, namely those in which the mechanism of synaptic clock has been successfully triggered, being presumably the same ones to which memory of a given experience has been potentially “allocated” (Rogerson et al., 2014). This could serve as a mechanism of noise reduction, contributing to what we perceive as a relatively stable flow of time.

## Brain Imaging Methods Allowing to Demonstrate a Link Between Temporal Perception Dynamics and the Postulated Functioning of Synaptic Clocks

Techniques of functional brain imaging which measure signals of the oxygenated blood flow (with magnetic resonance, fMRI, or near-infrared spectroscopy), can indicate an increase in activity of particular brain regions. It has been postulated that they are sensitive primarily to the presynaptic activity, i.e., the activity of afferents to a given site and magnitude of synaptic release, resulting in inputs to postsynaptic cells (Logothetis et al., 2001), which satisfies the definition of attention as used above. Therefore, these techniques might give a measure of a relative contribution of a particular brain region, with a specific population of synaptic clocks, to an overall, ongoing temporal perception. Indeed, there is evidence that increased activations of specific brain regions (i.e., right putamen, amygdala and insula), as indicated by fMRI, correlate with distortions of time perception (Dirnberger et al., 2012).

Techniques which measure field potentials generated by populations of neurons (electroencephalography (EEG) or local field potential recordings), as reflecting mostly synaptic currents (Haider et al., 2016), might directly demonstrate and support the notion and analogy of synaptic “CFF.” Namely, when a given neural population is working in synchrony, these techniques can indicate the frequency of its oscillating activity and rate of synaptic input to the postsynaptic cells, thereby showing the amount of information received by synapses in a unit time, which, as it seems, tends to correlate with the global dynamics of temporal perception. For instance: (1) a direct correlate of the CFF measure is found in EEG recorded over the visual cortex during observation of a flickering light stimulus, as Steady-State Visual Evoked Potentials, that is, field oscillations with a frequency corresponding to the frequency of a flicker (Kuś et al., 2013), which should correlate with alterations in the perceived speed of time flow when different amount of visual information is detected in a unit time, depending on the flicker frequency; (2) frequency of the theta rhythm in hippocampus correlates with the running speed of animal (Sławińska and Kasicki, 1998) and the running speed, in turn, should also correlate with the perceived speed of time flow, as the same amount of time is used to cover distances of different length; (3) the high-amplitude low-frequency oscillations in delta band (∼1 Hz) recorded in the cortex during quiescent states (Buzsáki, 1996) appear to speed up the perceived time flow, as less amount of information is processed in a unit time and time intervals seem to be shortened. Importantly, when the inter-stimulus interval (i.e., oscillation period) is shorter than the relevant time constants of postsynaptic clocks (see section “Synaptic Trace as a Dynamical Process”), inputs will be fused (a synaptic “flicker fusion effect”), however, when the interval is longer, its value will affect the number of changes detected by synapses in a unit time.

## Dependence of Temporal Perception on Neuromodulation

Neuromodulation, especially by DA, besides its role which can be viewed as a substitute of environmental feedbacks for synaptic activity (Schultz, 1997; Wang et al., 2010), can also influence an ongoing time perception, especially in emotional states (Paton and Buonomano, 2018). Specifically, it could exert its effect either by directly interfering with the dynamics of synaptic traces and thus functioning of synaptic clocks, or indirectly, by modulating the neuronal excitability, presynaptically relative to a given site, and thus altering the rate of synaptic input. Such a DA action could help explain the reported dependence of temporal perception on the midbrain DAergic neurons’ activity (Soares et al., 2016) and its distortions in pathological states affecting the DA system, e.g., in Parkinson’s disease patients (Mioni et al., 2018).

## Discussion

The extrapolation of the CFF property, specifying a temporal resolution of information processing, to brain functions other than visual perception may be justified by the studies of visual imagery. Namely, they show in humans that an overlapping neural substrate is activated both by an actual sensory experience and its subsequent explicit imagery (i.e., recall; Albright, 2012). Consequently, a detailed recall of past events is thought to be a reconstruction of previous states of the brain. Moreover, an implicit imagery is an integral part of any sensory experience, with sensory data being completed and interpreted in light of the contents of memory, accumulated over the lifetime, in order to form a meaningful mental scene. These observations suggest that direct sensory perception and, on the other hand, internal, autonomous mental states might be viewed as a sort of continuum and a property analogous to the CFF, as strictly dependent on memory, will apply to any of them.

It is usually assumed that a higher temporal resolution of information processing should be associated with higher energy expenditure per unit time and should correlate with higher rates of neural activity and metabolism (more “effort” put into processing) and, on the other hand, with more neural resources used to represent the information. I argue, however, that the resolution of a perceived information is determined primarily by a process which is essentially passive, and that a higher resolution is a result of shorter persistence (i.e., faster decay) of memory traces, as substantiated by the putative decay of memory traces of synaptic inputs. Shorter time units allow for a greater flexibility of neural activity and a higher number of distinct events to be processed in a unit time [which leads to shorter reaction times (Hagura et al., 2012)], but a higher resolution is hypothesized here to be related to the processing of information which is simpler (i.e., less abstract, direct sensory data), and such information should require less neural resources to be represented. Therefore, an increase in the amount of information per unit time will be compensated by a decrease in the complexity of this information. Indeed, the temporal resolution is shown to increase not with net, but rather with a mass-specific metabolic rate (Healy et al., 2013).

A standard, working memory-based model of interval timing, the Pacemaker-Accumulator Model, assumes an “accumulator” module which integrates “ticks” generated at a certain rate by a “pacemaker” module and subsequently stores them in the working memory for future reference (Buhusi and Meck, 2005). Then, when assessed retrospectively, the number of ticks accumulated over a given interval of absolute time gives a subjective measure of how much time that interval lasted. When the pacemaker is running faster more ticks are accumulated over an interval of the same length, affecting a subsequent estimate of its duration proportionally. It is assumed that ticks, generated by such a pacemaker and received by accumulator, are discrete events and temporal “size,” i.e., time of generation, of every tick is equal. I propose, in contrast, that a model of this kind could be refined by considering that durations of the ticks can vary, with every tick representing a different amount of absolute time, depending on what pacemaker they are generated by. The persistence of synapse-specific traces resulting in the E-LTP and transient amelioration of synaptic transmission likely alters the routes of information flow through the network, affecting thus the content of output projected from a given region. One could postulate that this content will change over time with a rate restricted by the rate of decay of synaptic traces, as represented by the time units of synaptic clocks, specific to a region. Then, neural activity coming out of different regions within a given interval of absolute time will carry information about a different, limited number of distinct “events,” or ticks, which could be then fed to an accumulator, with various brain regions being used acting as separate pacemakers. Different pacemakers of this type would be thus contributing ticks representing different amounts of absolute time as, depending on the pacemaker used, it will take a varied amount of time before a single tick is generated and accumulated. Eventually, when assessing the duration of an interval retrospectively, only the number of ticks accumulated can be counted, without any “access” to the amount of absolute time that each of them represents. This approach could help explain the variability of subjective judgments about durations of past intervals of absolute time, especially in situations when the performance of timing tasks required processing of streams of information generated by different systems (Muller and Nobre, 2014).

Although studies on the time course of persistence of synaptic tags, or so-called “eligibility traces,” in different brain regions have been sparse to date, there are certain pieces of direct and indirect evidence, coming mostly from rodent studies, which indicate that indeed the time window between synaptic activation and delivery of an effective feedback, which can lead to an enduring synaptic change, might vary depending on brain region. Particularly, it seems to be relatively long in the hippocampus (in the order of minutes) (Lisman and Grace, 2005), and shorter in the striatum (in the order of seconds) (Yagishita et al., 2014), which may support the existence of a distribution postulated here.

A short-term synaptic plasticity (STP), attributed primarily to the presynaptic processes of use-dependent facilitation or depression of transmitter release, has been proposed as an universal mechanism that can underlie emergence of neurons coding for various temporal properties of sensory stimuli over relatively constant timescales of tens to hundreds of milliseconds (Fortune and Rose, 2001; Motanis et al., 2018). It has been shown also that synapses both on post- as well as presynaptic cells can undergo a process of synapse-specific tagging and subsequent long-term facilitation (Frey and Morris, 1997; Martin et al., 1997; Martin, 2002), which occurs on timescales broader than those of the STP. Taken together, these observations show that transient, protein synthesis-independent processes of activity-dependent synaptic plasticity take place on the both ends of synaptic junction. Thus, the concept of synaptic clock could be thought of more generally as a basic functional entity on the level of single synapse, which results from the activity-dependent synaptic plasticity that can involve the both ends of synaptic junction, and which critically affects the perception of time over synapse-specific timescales that could be generally defined by their common feature as protein synthesis independent.

## Conclusion

In the present study I have shown how the properties of subjective perception of time may arise and evolve in different animal species, and how it could depend on the persistence of memory traces of previous inputs, with the synapse, and the proposed mechanism of synaptic clock based on a notion of synaptic tagging, being a plausible site where such operations are performed. I postulated the existence of a brain-wide distribution of time units of synaptic clocks, shaped evolutionarily by rate of interactions of organisms with their surroundings, and that variations in the perceived speed of time passage in humans are primarily due to the functioning of such a clocks measuring time in different units. This is thus a novel, biologically plausible, simple theoretical link between, on the one hand, the fields of time perception and synaptic-level memory, and, more generally, the levels of analysis of neuroscience and ecology.

## Data Availability

All datasets analyzed for this study are included in the manuscript and the supplementary files.

## Author Contributions

BJ conceived the theory and wrote the manuscript.

## Conflict of Interest Statement

The author declares that the research was conducted in the absence of any commercial or financial relationships that could be construed as a potential conflict of interest.

## References

[B1] Albright T. D. (2012). On the perception of probable things. neural substrates of associative memory, imagery and perception. *Neuron* 74 227–245. 10.1016/j.neuron.2012.04.001 22542178 PMC3361508

[B2] Autrum H. (1949). Neue versuche zum optischen auflösungsvermögen fliegender insekten. *Experientia* 7 271–277. 10.1007/bf02149940 18133506

[B3] Ballarini F. Moncada D. Martinez M. C. Alen N. Viola H. (2009). Behavioral tagging is a general mechanism of long-term memory formation. *Proc. Natl. Acad. Sci. U.S.A.* 106 14599–14604. 10.1073/pnas.0907078106 19706547 PMC2732837

[B4] Bliss T. V. Lømo T. (1973). Long-lasting potentiation of synaptic transmission in the dentate area of the anaesthetized rabbit following stimulation of the perforant path. *J. Physiol.* 232 331–356. 10.1113/jphysiol.1973.sp010273 4727084 PMC1350458

[B5] Boström J. E. Dimitrova M. Canton C. Håstad O. Qvarnström A. Ödeen A. (2016). Ultra-rapid vision in birds. *PLoS One* 11:e0151099. 10.1371/journal.pone.0151099 26990087 PMC4798572

[B6] Broussard J. I. Yang K. Levine A. T. Tsetsenis T. Jenson D. Cao F. (2016). Dopamine regulates aversive contextual learning and associated in vivo synaptic plasticity in the hippocampus. *Cell Rep.* 14 1930–1939. 10.1016/j.celrep.2016.01.070 26904943 PMC4772154

[B7] Buhusi C. V. Meck W. H. (2005). What makes us tick? *Nat. Rev. Neurosci.* 6 755–765. 10.1038/nrn1764 16163383

[B8] Buzsáki G. (1996). The hippocampo-neocortical dialogue. *Cereb. Cortex* 6 81–92. 10.1093/cercor/6.2.81 8670641

[B9] Díaz-Mataix L. Tallot L. Doyère V. (2014). The amygdala: a potential player in timing CS-US intervals. *Behav. Process.* 101 112–122. 10.1016/j.beproc.2013.08.007 23973708

[B10] Dirnberger G. Hesselmann G. Roiser J. P. Preminger S. Jahanshahi M. Paz R. (2012). Give it time: neural evidence for distorted time perception and enhanced memory encoding in emotional situations. *Neuroimage* 63 591–599. 10.1016/j.neuroimage.2012.06.041 22750720

[B11] Eagleman D. M. Pariyadath V. (2009). Is subjective duration a signature of coding efficiency? *Philos. Trans. R. Soc. B Biol. Sci.* 364 1841–1851. 10.1098/rstb.2009.0026 19487187 PMC2685825

[B12] Fortune E. S. Rose G. J. (2001). Short-term plasticity as a temporal filter. *Trends Neurosci.* 24 381–385. 10.1016/S0166-2236(00)01835-X 11410267

[B13] Frank T. M. (2000). Temporal resolution in mesopelagic crustaceans. *Philos. Trans. R. Soc. B Biol. Sci.* 355 1195–1198. 10.1098/rstb.2000.0666 11079397 PMC1692829

[B14] Frey U. Morris R. G. (1997). Synaptic tagging and long-term potentiation. *Nature* 385 533–536. 10.1038/385533a0 9020359

[B15] Gouvêa T. S. Monteiro T. Motiwala A. Soares S. Machens C. Paton J. J. (2015). Striatal dynamics explain duration judgments. *eLife* 4:e11386. 10.7554/eLife.11386 26641377 PMC4721960

[B16] Hagura N. Kanai R. Orgs G. Haggard P. (2012). Ready steady slow: action preparation slows the subjective passage of time. *Proc. R. Soc. B U.S.A.* 279 4399–4406. 10.1098/rspb.2012.1339 22951740 PMC3479796

[B17] Haider B. Schulz D. P. A. Häusser M. Carandini M. (2016). Millisecond coupling of local field potentials to synaptic currents in the awake visual cortex. *Neuron* 90 35–42. 10.1016/j.neuron.2016.02.034 27021173 PMC4826437

[B18] Hardy A. C. (1920). A study of the persistence of vision. *Proc. Natl. Acad. Sci. U.S.A.* 6 221–224. 16576494 10.1073/pnas.6.4.221 PMC1084467

[B19] Healy K. McNally L. Ruxton G. D. Cooper N. Jackson A. L. (2013). Metabolic rate and body size are linked with perception of temporal information. *Anim. Behav.* 86 685–696. 10.1016/j.anbehav.2013.06.018 24109147 PMC3791410

[B20] Huang Y. Y. Simpson E. Kellendonk C. Kandel E. R. (2004). Genetic evidence for the bidirectional modulation of synaptic plasticity in the prefrontal cortex by D1 receptors. *Proc. Natl. Acad. Sci. U.S.A.* 101 3236–3241. 10.1073/pnas.0308280101 14981263 PMC365773

[B21] Jékely G. (2009). Evolution of phototaxis. *Phil. Trans. R. Soc. B* 364 2795–2808. 10.1098/rstb.2009.0072 19720645 PMC2781859

[B22] Kanai R. Paffen C. L. Hogendoorn H. Verstraten F. A. (2006). Time dilation in dynamic visual display. *J. Vis.* 6 1421–1430. 10.1167/6.12.8 17209745

[B23] Kuś R. Duszyk A. Milanowski P. Łabȩcki M. Bierzyńska M. Radzikowska Z. (2013). On the quantification of SSVEP frequency responses in human EEG in realistic BCI conditions. *PLoS One* 8:e77536. 10.1371/journal.pone.0077536 24204862 PMC3799619

[B24] Lake J. I. LaBar K. S. Meck W. H. (2016). Emotional modulation of interval timing and time perception. *Neurosci. Biobehav. Rev.* 64 403–420. 10.1016/j.neubiorev.2016.03.003 26972824 PMC5380120

[B25] Lee S. J. R. Escobedo-Lozoya Y. Szatmari E. M. Yasuda R. (2009). Activation of CaMKII in single dendritic spines during long-term potentiation. *Nature* 458 299–304. 10.1038/nature07842 19295602 PMC2719773

[B26] Lengyel I. Voss K. Cammarota M. Bradshaw K. Brent V. Murphy K. P. (2004). Autonomous activity of CaMKII is only transiently increased following the induction of long-term potentiation in the rat hippocampus. *Eur. J. Neurosci.* 20 3063–3072. 10.1111/j.1460-9568.2004.03748.x 15579161

[B27] Lewis P. A. Miall R. C. (2003). Distinct systems for automatic and cognitively controlled time measurement: evidence from neuroimaging. *Curr. Opin. Neurobiol.* 13 250–255. 10.1016/S0959-4388(03)00036-9 12744981

[B28] Lisman J. Grace A. A. (2005). The hippocampal-VTA loop: controlling the entry of information into long-term memory. *Neuron* 46 703–713. 10.1016/j.neuron.2005.05.002 15924857

[B29] Lisman J. Grace A. A. Duzel E. (2001). A neoHebbian framework for episodic memory: role of dopamine-dependent late LTP. *Trends Neurosci.* 34 536–547. 10.1016/j.tins.2011.07.006 21851992 PMC3183413

[B30] Lisman J. Yasuda R. Raghavachari S. (2012). Mechanisms of CaMKII action in long term potentiation. *Nat. Rev. Neurosci.* 13 169–182. 10.1038/nrn3192 22334212 PMC4050655

[B31] Lisney T. J. Rubene D. Rózsa J. Løvlie H. Håstad O. Ödeen A. (2011). Behavioural assessment of flicker fusion frequency in chicken Gallus gallus domesticus. *Vision Res.* 51 1324–1332. 10.1016/j.visres.2011.04.009 21527269

[B32] Logothetis N. K. Pauls J. Augath M. Trinath T. Oeltermann A. (2001). Neurophysiological investigation of the basis of the fMRI signal. *Nature* 412 150–157. 10.1038/35084005 11449264

[B33] Martin K. C. (2002). Synaptic tagging during synapse-specific long-term facilitation of *Aplysia* sensory-motor neurons. *Neurobiol. Learn. Mem.* 78 489–497. 10.1006/nlme.2002.4088 12559829

[B34] Martin K. C. Casadio A. Zhu H. Yaping E. Rose J. C. Chen M. (1997). Synapse-specific, long-term facilitation of *Aplysia* sensory to motor synapses: a function for local protein synthesis in memory storage. *Cell* 91 927–938. 10.1016/S0092-8674(00)80484-5 9428516

[B35] Melcher D. (2001). Persistence of visual memory for scenes. *Nature* 412:401. 10.1038/35086646 11473303

[B36] Mioni G. Capizzi M. Vallesi A. Correa A. Di Giacopo R. Stablum F. (2018). Dissociating explicit and implicit timing in Parkinson’s disease patients: evidence from bisection and foreperiod tasks. *Front. Hum. Neurosci.* 12:17. 10.3389/fnhum.2018.00017 29467632 PMC5808217

[B37] Moncada D. Ballarini F. Martinez M. C. Frey J. U. Viola H. (2011). Identification of transmitter systems and learning tag molecules involved in behavioral tagging during memory formation. *Proc. Natl. Acad. Sci. U.S.A.* 108 12931–12936. 10.1073/pnas.1104495108 21768371 PMC3150922

[B38] Motanis H. Seay M. J. Buonomano D. V. (2018). Short-term synaptic plasticity as a mechanism for sensory timing. *Trends Neurosci.* 41 701–711. 10.1016/j.tins.2018.08.001 30274605 PMC6171349

[B39] Muller T. Nobre A. C. (2014). Perceiving the passage of time: neural possibilities. *Ann. N. Y. Acad. Sci.* 1326 60–71. 10.1111/nyas.12545 25257798 PMC4336553

[B40] Nardella A. Rocchi L. Conte A. Bologna M. Suppa A. Berardelli A. (2014). Inferior parietal lobule encodes visual temporal resolution processes contributing to the critical flicker frequency threshold in humans. *PLoS One* 9:e98948. 10.1371/journal.pone.0098948 24905987 PMC4048231

[B41] Okamoto K. Bosch M. Hayashi Y. (2009). The roles of CaMKII and F-actin in the structural plasticity of dendritic spines: a potential molecular identity of a synaptic tag? *Physiology* 24 357–366. 10.1152/physiol.00029.2009 19996366

[B42] Otani S. Bai J. Blot K. (2015). Dopaminergic modulation of synaptic plasticity in rat prefrontal neurons. *Neurosci. Bull.* 31 183–190. 10.1007/s12264-014-1507-3 25822215 PMC5563702

[B43] Paton J. J. Buonomano D. V. (2018). The neural basis of timing: distributed mechanisms for diverse functions. *Neuron* 98 687–705. 10.1016/j.neuron.2018.03.045 29772201 PMC5962026

[B44] Reynolds J. N. Wickens J. R. (2002). Dopamine-dependent plasticity of corticostriatal synapses. *Neural Netw.* 15 507–521. 10.1016/S0893-6080(02)00045-X 12371508

[B45] Rogerson T. Cai D. Frank A. Sano Y. Shobe J. Aranda M. L. (2014). Synaptic tagging during memory allocation. *Nat. Rev. Neurosci.* 15 157–169. 10.1038/nrn3667 24496410 PMC3992944

[B46] Sajikumar S. Navakkode S. Frey J. U. (2007). Identification of compartment- and process- specific molecules required for “synaptic tagging” during long-term potentiation and long-term depression in hippocampal CA1. *J. Neurosci.* 27 5068–5080. 10.1523/jneurosci.4940-06.2007 17494693 PMC6672381

[B47] Schmitz O. (2017). Predator and prey functional traits: understanding the adaptive machinery driving predator-prey interactions. *F1000Res* 6:1767. 10.12688/f1000research.11813.1 29043073 PMC5621104

[B48] Schuergers N. Lenn T. Kampmann R. Meissner M. V. Esteves T. Temerinac-Ott M. (2016). Cyanobacteria use micro-optics to sense light direction. *eLife* 5:e12620. 10.7554/eLife.12620 26858197 PMC4758948

[B49] Schultz W. (1997). Dopamine neurons and their role in reward mechanisms. *Curr. Opin. Neurobiol.* 7 191–197. 10.1016/S0959-4388(97)80007-4 9142754

[B50] Sławińska U. Kasicki S. (1998). The frequency of rat’s hippocampal theta rhythm is related to the speed of locomotion. *Brain Res.* 796 327–331. 10.1016/S0006-8993(98)00390-4 9689489

[B51] Soares S. Atallah B. V. Paton J. J. (2016). Midbrain dopamine neurons control judgment of time. *Science* 354 1273–1277. 10.1126/science.aah5234 27940870

[B52] Sweatt J. D. (2016). Neural plasticity and behavior – sixty years of conceptual advances. *J. Neurochem.* 139 179–199. 10.1111/jnc.13580 26875778

[B53] Thivierge J. P. Rivest F. Monchi O. (2007). Spiking neurons, dopamine, and plasticity: timing is everything, but concentration also matters. *Synapse* 61 375–390. 10.1002/syn.20378 17372980

[B54] Umeton D. Read J. D. A. Rowe C. (2017). Unravelling the illusion of flicker fusion. *Biol. Lett.* 13:20160831. 10.1098/rsbl.2016.0831 28148834 PMC5326512

[B55] Wadhams G. Armitage J. P. (2004). Making sense of it all: bacterial chemotaxis. *Nat. Rev. Mol. Cell Biol.* 5 1024–1037. 10.1038/nrm1524 15573139

[B56] Wang S. H. Redondo R. L. Morris R. G. (2010). Relevance of synaptic tagging and capture to the persistence of long-term potentiation and everyday spatial memory. *Proc. Natl. Acad. Sci. U.S.A.* 107 19537–19542. 10.1073/pnas.1008638107 20962282 PMC2984182

[B57] Yagishita S. Hayashi-Takagi A. Ellis-Davies G. C. R. Urakubo H. Ishii S. Kasai H. (2014). A critical time window for dopamine actions on the structural plasticity of dendritic spines. *Science* 345 1616–1620. 10.1126/science.1255514 25258080 PMC4225776

